# The association among calorie, macronutrient, and micronutrient intake with colorectal cancer: A case–control study

**DOI:** 10.1002/fsn3.2775

**Published:** 2022-04-20

**Authors:** Maryam Gholamalizadeh, Mojgan Behrad Nasab, Mina Ahmadzadeh, Saeid Doaei, Mona Jonoush, Soheila Shekari, Maryam Afsharfar, Payam Hosseinzadeh, Saheb Abbastorki, Mohammad Esmail Akbari, Maryam Hashemi, Saeed Omidi, Farhad Vahid, Alireza Mosavi Jarrahi, Ali Lavasani

**Affiliations:** ^1^ 556492 Cancer Research Center Shahid Beheshti University of Medical Sciences Tehran Iran; ^2^ Department of Physical Education & Sport Sciences Faculty of Sport Science Central Tehran Branch Islamic Azad University Tehran Iran; ^3^ 556492 Department of Clinical Nutrition and Dietetics Faculty of Nutrition and Food Technology National Nutrition and Food Technology Research Institute Shahid Beheshti University of Medical Sciences Tehran Iran; ^4^ 37554 School of Health, Research Center of Health and Environment Guilan University of Medical Sciences Rasht Iran; ^5^ Department of Nutrition School of Medicine Mashahd University of Medical Sciences Mashahad Iran; ^6^ Department of Nutrition, Science and Research Branch Islamic Azad University Tehran Iran; ^7^ Department of Nutrition School of Medicine Zahedan University of Medical Sciences Zahedan Iran; ^8^ Gastrointestinal and liver Diseases Research Center (GLDRC), Iran University of Medical Sciences Tehran Iran; ^9^ Department of Nutrition Faculty of Nutrition Sciences Shiraz University of Medical Sciences Shiraz Iran; ^10^ Department of Pathology Firoozgar General Hospital Iran University of Medical Sciences Tehran Iran; ^11^ 37554 Department of Health Education and Promotion School of Health Research Center of Health and Environment Guilan University of Medical Sciences Rasht Iran; ^12^ 58942 Nutrition and Health Research Group Department of Population Health Luxembourg Institute of Health Strassen Luxembourg; ^13^ 556492 School of Medicine Shahid Beheshti University of Medical Sciences Tehran Iran

**Keywords:** colorectal cancer, dietary intake, nutrients

## Abstract

The risk of colorectal cancer (CRC) can be influenced by dietary components. This study aims to investigate the association between dietary intake and CRC in Iranian adults. This hospital‐based case–control study was performed on 160 patients with CRC and 320 healthy people. General and pathological data were collected through face‐to‐face interviews. A validated food frequency questionnaire (FFQ) was used to assess the intake of calories, macronutrients, and micronutrients. The case group had a significantly higher intake of calories, carbohydrates, vitamin A, vitamin K, fluoride, and molybdenum and a lower intake of vitamin E, vitamin B1, beta carotene, biotin, folate, magnesium, selenium, manganese, and fiber (all *p* < .001). CRC was positively associated with the intake of carbohydrate (OR: 1.01, CI% 1.03–1.01, *p* = .001), and vitamin A (OR: 1.009, CI 95% 1.006–1.01, *p* = .001) and negatively associated with intake of fiber (OR: 0.67, CI 95% 0.59–0.76, *p* = .001), beta carotene (OR: 0.99, CI 95% 0.99–0.99, *p* = .001), vitamin E (OR: 0.27, CI 95% 0.15–0.47, *p* = .001), folate (OR: 0.98 CI 95% 0.97–0.98, *p* = .001), and biotin (OR: 0.83, CI 95% 0.77–0.90, *p* = .001). The associations remained significant after adjusting for age and sex. Further adjustments for physical activity, alcohol consumption, and smoking did not change the results. The results identified that the risk of colorectal cancer can be influenced by dietary intake. Further longitudinal studies are needed to confirm these findings and to identify the underlying mechanisms of the effects of dietary components on the risk of colorectal cancer.

## Graphical abstract

The results of this study indicated that the CRC patients had a significantly higher intake of calorie, carbohydrates, vitamin A, vitamin K, fluoride, and molybdenum and a lower intake of vitamin E, beta carotene, biotin, folate, magnesium, selenium, manganese, and fiber. There was a positive association between CRC and dietary intake of carbohydrate and vitamin A and a negative association with the intake of fiber, beta carotene, vitamin E, folate, and biotin.

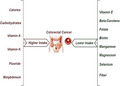

## 1 INTRODUCTION

Colorectal cancer (CRC) is the second most prevalent cancer and the fourth leading cause of cancer death in the world (Rafiemanesh et al., 2016). The incidence of CRC is increasing in recent decades and the World Health Organization (WHO) reported that the number of new cases and death of colon and rectum cancer was 1.93 million and 935,000, respectively (Bray et al., 2018). However, the mortality rate of CRC has been recently reduced mainly due to effective cancer screening programs (Thanikachalam & Khan, 2019). CRC in Iranian females and males is the second and third most prevalent cancer, accounting for 7 and 8 per 100 thousand people, respectively (Rezapour et al., 2021).

Several factors may have a role in the risk of CRC such as genetics, history of inflammatory bowel disease, unhealthy diet, and physical inactivity (Doaei et al., 2019, 2021; Dolatkhah et al., 2015). An unbalanced diet such as a high‐calorie and high‐fat diet was reported to increase the risk of CRC (Mehrdad et al., 2020; Ströhle et al., 2007). In one study, the relative risk of CRC was 49.2% higher in women who ate beef, pork, or lamb as their main daily food compared to eating meat less than once a month (Levin, 1992). In addition, consumption of red meat was associated with an increase in CRC compared with chicken and fish (Levin, 1992; Norat et al., 2005).

On the other hand, some nutrients such as dietary fiber, fats, and calcium were reported to have a role in the prevention of CRC (Levin, 1992; Potter, 1996). Fruits and vegetables have antioxidant properties including carotenoids, flavonoids, selenium, vitamin C, vitamin E, and plant sterols, which may play important roles in reducing the risk of CRC (Levin, 1992). One study found that doubling dietary fiber intake in populations with low fiber intake reduced the risk of CRC by up to 40% (Bingham et al., 2003). The effect of folate supplementation on colorectal dysplasia confirmed the positive relationship between folate deficiency and DNA hypomethylation with CRC (Levin, 1992). Therefore, a diet rich in folate may reduce the risk of CRC (Ströhle et al., 2007; Willett, 2000). Furthermore, dietary calcium may play a protective role for CRC by affecting cell proliferation and tumor induction (Levin, 1992). Some study reported that consuming milk and dairy products decrease the risk of CRC (Ströhle et al., 2007).

However, conflicting results were reported on the association of some dietary components and CRC. For example, a recent review study failed to establish a link between dietary fiber and the risk of CRC (Doyle, 2007). Furthermore, another study reported that antioxidant vitamins including vitamin E, vitamin C, beta carotene, and flavonoids did not reduce oxidative DNA damage and had no anticancer effect (Halliwell, 2002). Due to the contradictory results in the previous studies, this case–control study aimed to investigate the association of calorie, macronutrient, and micronutrient intake and CRC in Iranian adults.

## 2 METHODS

### 2.1 Study population

This hospital‐based case–control study was conducted on 160 patients with CRC as the case group and 320 healthy people as the control group who were randomly selected from those referred to three hospitals (i.e., Firoozgar, Shohadaye Tajrish, and Shahid Taleghani hospitals) of Tehran, Iran, between June 2020 and March 2021. The inclusion criteria of the case group included a willingness to participate in the study, confirmed histopathologic CRC, a maximum of 2 months elapsed since primary diagnosis, and an age range of 35 to 70 years. Inclusion criteria of the control group included a willingness to participate in this study, having no malignancy, and an age range of 35 to 70 years.

Demographic variables including age, gender, marital status, and ethnicity were collected through a face‐to‐face interview. The measurements of weight and height were done by trained researchers. Height was measured using a portable stadiometer (Seca 213), without shoes, and recorded to the nearest 0.1 cm. Height was measured with the head of participants in anatomical position, knees straight, and the heels, buttocks, and the shoulders blades touching the vertical surface of the Stadiometer. The patient's weight was measured using a SECA Alpha 882 scale (SECA Corporation) and BMI was calculated based on weight divided by height squared (Kirk et al., 2009). A validated international physical activity questionnaire (IPAQ) was used to measure participants' physical activity (Vasheghani‐Farahani et al., 2011). Results obtained from IPAQ were presented as metabolic equivalents (METS) per minute. The protocol of the study was approved by the Ethics Committee of Shahid Beheshti University of Medical Sciences, Tehran, Iran (code: IR.SBMU.CRC.REC.1398.028). The objectives of the study were explained to the participants and informed written consent was obtained.

### 2.2 Dietary assessment

The dietary intake of the participants was assessed through face‐to‐face interviews by a trained dietitian using a validated 168‐item Semi‐Quantitative Food Frequency Questionnaire (FFQ) consisting of 168 food items with standard portion sizes commonly consumed by Iranian people (Mirmiran et al., 2010). The collected data on dietary intake was analyzed using the Nutritionist‐IV software (N Squared Computing) (Azar & Sarkisian, 1980). Daily intakes of calories, macronutrients, and micronutrients were calculated for each participant by using the US Department of Agriculture food consumption database, which was modified for Iranian foods (Haytowitz, Ahuja et al., 2018).

### 2.3 Statistical analysis

Hardy–Weinberg equilibrium was used for the evaluation of genotype distribution. General characteristics of the case and control groups were compared by Chi‐square (for qualitative variables) and independent *t*‐test (for quantitative variables) methods. Normal distribution of data was assessed using the Shapiro–Wilk test. Binary logistic regression analysis was performed, and regression models were fitted to investigate the association between CRC and the dietary intake as crude (model 1), after adjusting for age and sex (model 2), further adjustments for smoking and alcohol consumption (model 3), and additional adjustments for BMI (model 4). Data were analyzed using statistical software (IBM Inc.). Two‐sided statistical tests were used, and *p* < .0012 was considered significant after Bonferroni correction for multiple comparisons.

## 3 RESULTS

The data were normally distributed. Demographic and anthropometric characteristics of the case and control groups are presented in Table 1. The cases had higher height (158.49 ± 7.59 vs. 156.11 ± 5.31 cm, *p* = .001), lower BMI (27.58 ± 3.25 vs. 28.76 ± 3.97 kg/m^2^, *p* = .001), and higher smoking (7.6% vs. 0.8%, *p* = .003) compared to the control groups. There was no significant difference between the two groups in terms of age, sex, weight, physical activity, and alcohol consumption (Table 2).

**TABLE 1:** Distribution of characteristics of the participants (*N* = 480)

Variables	Mean ± SD or *N* (%)		*p*‐Value
	Cases (*n* = 160)	Controls (*n* = 320)	
Age (year)	52.36 ± 17.06	51.8 ± 10.82	.06
Males (*n*, %)	81 (54.7%)	163 (51.1%)	.11
Weight (kg)	69.39 ± 8.64	70.12 ± 10.59	.44
Height (Cm)	158.49 ± 7.59	156.11 ± 5.31	<.001
BMI (kg/m^2^)	27.58 ± 3.25	28.76 ± 3.97	<.001
Physical activity (h/week)	7.51 ± 1.95	7.31 ± 1.58	.28
Smoking (*n*, %)	12 (7.6%)	3 (0.9%)	<.01
Alcohol use (*n*, %)	0 (0.0%)	3 (1.2%)	.73

**TABLE 2:** Dietary intake of the participants (*N* = 480)

Variables	Mean ± SD		*p*‐Value	Median (IQR)^a^	
	Cases (*n* = 160)	Controls (*n* = 320)		Cases (*n* = 160)	Controls (*n* = 320)
Calorie (kcal/day)	2568.76 ± 404.48	2493.38 ± 176.03	<.00	2536.78 (49.1)	2546.62 (45.8)
Protein (g/day)	85.77 ± 9.16	85.39 ± 19.85	.78	86.11 (9.91)	85.44 (9.07)
Carbohydrate (g/day)	368.88 ± 51.49	354.28 ± 33.72	<.001	361.29 (19.19)	364.63 (17.08)
Fat (g/day)	88.90 ± 10.62	90.75 ± 20.15	.20	90.07 (9.41)	89.96 (9.86)
Sodium (mg/day)	6355.03 ± 1523.70	6085.03 ± 1059.26	.06	6160.87 (88.11)	6164.56 (96.62)
Potassium (mg/day)	3921.24 ± 389.23	3990.76 ± 886.81	.38	3962.25 (74.04)	3970.63 (70.57)
Iron (mg/day)	18.71 ± 3.1	18.62 ± 3.1	.78	18.77 (3.66)	18.74 (3.34)
Calcium (mg/day)	1206.96 ± 118.71	1230.07 ± 498.98	.44	1221.10 (54.87)	1219.44 (53.42)
Magnesium (mg/day)	332.02 ± 42.90	348.88 ± 73.66	<.001	342.34 (20.61)	344.70 (22.12)
Phosphorus (mg/day)	1396.36 ± 172.87	1412.65 ± 461.03	.58	1409.49 (58.61)	1400.15 (61.10)
Zinc (mg/day)	10.75 ± 2.44	10.59 ± 3.43	.59	10.98 (3.65)	10.32 (3.66)
Copper (mg/day)	1.56±0.66	1.68±0.69	.10	1.51 (0.91)	1.68 (1.07)
Manganese (mg/day)	4.83 ± 1.74	5.2 ± 1.91	.02	4.63 (2.25)	5.08 (2.44)
Selenium (mg/day)	51.54 ± 25.96	68.70 ± 20.13	<.001	61.51 (14.65)	64.52 (12.72)
Fluoride (mg/day)	15,831.32 ± 9773.24	10,820.81 ± 3431.17	<.001	12,378.43 (230.64)	12,297.93 (209.96)
Chromium (mcg/day)	0.124±0.17	0.099±0.15	.15	0.015 (0.24)	0.003 (0.16)
Molybdenum (mcg/day)	50.89 ± 9.35	50.75 ± 4.70	<.001	50.81 (5.13)	50.70 (6.28)
Vitamin A (mcg/day)	842.47 ± 288.87	691.08 ± 158.84	<.001	741.90 (42.72)	731.09 (38.98)
Vitamin DAY (Mg/day)	1.04 ± 0.82	1.01 ± 0.77	.71	0.92 (1.06)	0.92 (1.13)
Vitamin K (Mg/day)	161.7 ± 48.95	144.01 ± 26.35	<.001	149.77 (14.60)	148.76 (14.13)
Vitamin E (Mg/day)	10.15 ± 4.16	13.10 ± 5.32	<.001	11.00 (5.84)	12.61 (4.84)
Vitamin B1 (mg/day)	2.01 ± 0.81	2.19 ± 0.84	.030	1.81 (0.99)	2.17 (1.10)
Vitamin B2 (mg/day)	2.23 ± 0.99	2.34 ± 1.18	.29	2.07 (1.32)	2.09 (1.53)
Vitamin B3 (mg/day)	21.44 ± 2.53	21.75 ± 3.14	.27	21.84 (3.05)	21.61 (3.69)
Vitamin B6 (mg/day)	1.93 ± 0.81	1.92 ± 0.847	.88	1.83 (1.01)	1.85 (1.19)
Folate (mg/day)	516.65 ± 96.59	571.05 ± 80.21	<.001	550.13 (25.09)	555.74 (24.22)
Vitamin B12 (mg/day)	4.47 ± 1.93	4.27 ± 2.51	.35	4.43 (2.89)	4.11 (2.69)
Vitamin B5 (mg/day)	5.51 ± 1.77	5.36 ± 1.93	.41	5.40 (2.19)	5.35 (2.32)
Biotin (mcg/day)	26.90 ± 4.58	28.76 ± 8.04	.001	27.21 (5.66)	28.33 (6.62)
Vitamin C (mg/day)	145.1 ± 21.7	150.97 ± 56.75	.11	150.34 (18.92)	148.64 (17.36)
Beta carotene	2076.30 ± 591.56	2468.07 ± 938.27	<.001	2073 (42)	2465 (46)
Fiber (g/day)	23.77 ± 4.86	26.01 ± 6.17	<.001	24.75 (5.41)	25.65 (5.64)

^a^ Interquartile range.

The dietary intake of the groups is presented in Table 3. The case group had significantly higher intake of calorie (2568.76 ± 404.48 vs. 2493.38 ± 176.03 kcal/day), carbohydrates (368.88 ± 51 vs. 354.28 ± 33.72 g/day), vitamin A (842.47 ± 288.87 vs. 691.08 ± 158.84 mcg), vitamin K (161.7 ± 48.95 vs. 144.01 ± 26.35 mcg), fluoride (15,831.32 ± 9773.24 vs. 10,820.81 ± 3431.17 mg/day), and molybdenum (50.89 ± 9.35 vs. 50.75 ± 4.70 mg/day), and lower intake of vitamin E (10.15 ± 4.16 vs. 13.10 ± 5.32 mg/day), biotin (26.90 ± 4.58 vs. 28.76 ± 8.04 mg/day), folate (516.65 ± 96.59 vs. 571.05 ± 80.21 mg/day), magnesium (4.83 ± 1.74 vs. 5.2 ± 1.91 mg/day), selenium (51.54 ± 25.96 vs. 68.70 ± 20.13 mg/day), biotin (26.90 ± 4.58 vs. 28.76 ± 8.04 mg/day), beta carotene (2076.30 ± 591.56 vs. 2468.07 ± 938.27 mg/day), and fiber (23.77 ± 4.86 vs. 26.01 ± 6.17 g/day) (all *p* < .001). There was no significant difference in the mean intake of fat, protein, vitamins B1, B2, B3, B5, B6, B12, C, and D, iron, calcium, zinc, chromium, manganese, copper, sodium, and potassium.

**TABLE 3:** Distribution (number %) of participants with values below/above the Dietary Reference Intakes

Variables	DRI values^a^	Cases (*n* = 136)		Controls (*n* = 314)		*p*‐Value^b^	Total (*N* = 450)	
		*N* (%) ˂ DRI	*N* (%) ≥ DRI	*N* (%) ˂ DRI	*N* (%) ≥ DRI		*N* (%) ˂ DRI	*N* (%) ≥ DRI
Sodium	2300 (mg/day)	0 (0%)	136 (100%)	1 (0.4%)	313 (99.6%)	.999	1 (0.3%)	449 (99.7%)
Potassium	4700 (mg/day)	133 (97.8%)	3 (2.2%)	303 (96.5%)	11 (3.5%)	.998	436 (96.8%)	14 (3.1%)
Iron	18 (mg/day)	53 (38.9%)	83 (61.1%)	123 (39.2%)	191 (60.8%)	.478	176 (39.1%)	274 (60.8%)
Calcium	1000 (mg/day)	8 (5.8%)	128 (94.2%)	21 (6.6%)	293 (93.4%)	.625	29 (6.4%)	421 (93.6%)
Magnesium	420 (mg/day)	134 (98.5%)	2 (1.5%)	301 (95.8%)	13 (4.2%)	.720	435 (96.6%)	15 (3.4%)
Phosphorus	700 (mg/day)	1 (0.7%)	135 (99.3%)	1 (0.4%)	313 (99.6%)	.999	2 (0.5%)	448 (99.5%)
Zinc	11 (mg/day)	68 (50%)	68 (50%)	188 (59.8%)	126 (40.1%)	.728	256 (56.8%)	194 (43.1%)
Copper	1100 (mg/day)	36 (26.4%)	100 (73.6%)	78 (24.8%)	236 (75.2%)	.810	114 (25.3%)	336 (74.7%)
Manganese	3 (mg/day)	17 (12.5%)	119 (87.5%)	29 (9.2%)	285 (90.8%)	.229	46 (10.2%)	404 (89.7%)
Selenium	55 (mg/day)	40 (29.4%)	96 (70.6%)	30 (9.4%)	284 (90.6%)	.349	70 (15.5%)	380 (84.4%)
Chromium	350 (mcg/day)	114 (83.8%)	22 (16.2%)	286 (91.1%)	28 (8.9%)	.998	400 (88.8%)	50 (11.2%)
Vitamin A	900 (mcg/day)	114 (83.8%)	22 (16.2%)	311 (99.1%)	3 (0.9%)	.164	425 (94.4%)	25 (5.6%)
Vitamin D	5 (mcg/day)	38 (28%)	98 (72%)	98 (31.2%)	216 (68.8%)	.305	136 (30.2%)	314 (69.7%)
Vitamin K	120 (mcg/day)	4 (2.9%)	132 (97.1%)	32 (10.2%)	282 (89.8%)	.999	36 (8%)	414 (92%)
Vitamin E	15 (mg/day)	121 (88.9%)	15 (11.1%)	233 (74.2%)	81 (25.8%)	.241	354 (78.6%)	96 (21.4%)
Vitamin B1	1.1 (mg/day)	21 (15.4%)	115 (84.6%)	34 (10.9%)	280 (89.1%)	.225	55 (12.2%)	395 (87.8%)
Vitamin B2	1.2 (mg/day)	26 (19.1%)	110 (80.9%)	63 (20.1%)	251 (79.9%)	.999	89 (19.7%)	361 (80.3%)
Vitamin B3	15 (mg/day)	4 (2.9%)	132 (97.1%)	7 (2.2%)	307 (97.8%)	.998	11 (2.4%)	439 (97.6%)
Vitamin B6	1.3 (mg/day)	32 (23.5%)	104 (76.5%)	80 (25.4%)	234 (74.6%)	.101	112 (24.8%)	338 (75.2%)
Folate	400 (mcg/day)	18 (13.2%)	118 (86.8%)	2 (0.6%)	312 (99.4%)	.999	20 (4.4%)	430 (95.6%)
Vitamin B12	2.4 (mcg/day)	19 (13.9%)	117 (86.1%)	58 (18.5%)	256 (81.5%)	.762	77 (17.1%)	373 (82.9%)
Vitamin B5	5 (mg/day)	55 (40.4%)	81 (59.6%)	136 (43.3%)	178 (56.7%)	.383	191 (42.4%)	259 (57.6%)
Biotin	30 (mcg/day)	106 (77.9%)	30 (22.1%)	207 (65.9%)	107 (34.1%)	.988	313 (69.5%)	137 (30.5%)
Vitamin C	85 (mg/day)	4 (2.9%)	132 (97.1%)	10 (3.2%)	304 (96.8%)	.143	14 (3.1%)	436 (96.9%)

^a^ Dietary Reference Intakes = DRI (U.S. DEPARTMENT OF AGRICULTURE; National Agricultural Library); {Available here: https://www.nal.usda.gov/legacy/fnic/dri‐nutrient‐reports}.

^b^ Chi‐square was used for comparing groups.

The number (%) of participants with values below/above the Dietary Reference Intakes (DRIs) and a comparison of micronutrient intake between the two groups are presented in Table 3. There was no significant difference in the number of people with micronutrients intake of above DRIs between the two groups. Most people in both groups consumed less than the DRI for potassium, magnesium, chromium, vitamin A, vitamin E, and biotin.

The association between dietary intake and the risk of colorectal cancer is presented in Table 4. CRC was positively associated with the intake of carbohydrate (OR: 1.01, CI% 1.03–1.01, *p* = .001) and vitamin A (OR: 1.009, CI 95% 1.006–1.01, *p* = .001) and negatively associated with intake of fiber (OR: 0.67, CI 95% 0.59–0.76, *p* = .001), vitamin E (OR: 0.27, CI 95% 0.15–0.47, *p* = .001), folate (OR: 0.98 CI 95% 0.97–0.98, *p* = .001), and biotin (OR: 0.83, CI 95% 0.77–0.90, *p* = .001) (Model 1). The associations remained significant after adjusting for age and sex (Model 2), and after further adjustments for physical activity, alcohol consumption, and smoking (model 3). Additional adjustments for BMI did not change the results (Model 4).

**TABLE 4:** Logistic regression of the association between colorectal cancer and dietary intakes

Variables	Model 1		Model 2		Model 3		Model 4	
	OR (CI 95%)	*p*‐Value	OR (CI 95%)	*p*‐Value	OR (CI 95%)	*p*‐Value	OR (CI 95%)	*p*‐Value
Calorie	1.01 (1.00–1.02)	.025	1.01 (1.00–1.02)	.022	1.03 (1.01–1.05)	.001	1.02 (1.01–1.04)	.111
Carbohydrate	1.01 (1.03–1.01)	.001	0.97 (0.95−0.98)	.001	1.02 (1.01–1.03)	.001	1.02 (1.02–1.03)	.001
Fiber	0.67 (0.59−0.76)	.001	0.70 (0.61–0.81)	.001	0.71 (0.61–0.83)	.001	0.72 (0.62–0.83)	.001
Vitamin A	1.01 (1.01–1.01)	.001	1.01 (1.01–1.01)	.001	1.01 (1.01–1.01)	.001	1.01 (1.01–1.01)	.001
Vitamin E	0.27 (0.15–0.47)	.001	0.29 (0.15–0.55)	.001	0.25 (0.11−0.57)	.001	0.28 (0.13–0.61)	.001
Vitamin B1	0.36 (0.19–0.69)	.002	0.27 (0.11–0.71)	.007	0.26 (0.09–0.68)	.006	0.26 (0.09–0.68)	.006
Folate	0.98 (0.97–0.98)	.001	0.98 (0.97–0.98)	.001	0.98 (0.97−0.098)	.001	0.98 (0.97−0.98)	.001
Biotin	0.83 (0.77–0.90)	.001	0.81 (0.73−0.90)	.001	0.81 (0.73–0.90)	.001	0.85 (0.76–0.95)	.001
Magnesium	0.97 (0.96–0.98)	.001	0.98 (0.97–0.98)	.001	0.98 (0.97–0.99)	.001	0.98 (0.97–0.99)	.001
Fluoride	1.01 (1.01–1.01)	.001	1.01 (1.00–1.01)	.121	1.01 (1.00–1.01)	.006	1.01 (0.41–2.5)	.971

Note

Model 1: Crude, Model 2: Adjusted for sex and age, Model 3: Further adjustments for sleep, smoking, and alcohol consumption, Model 4: Further adjustments for BMI.

## 4 DISCUSSION

The results of this study indicated that the CRC patients had a significantly higher intake of calorie, carbohydrates, vitamin A, vitamin K, fluoride, and molybdenum and a lower intake of vitamin E, beta carotene, biotin, folate, magnesium, selenium, manganese, and fiber. There was no significant difference in the mean intake of vitamins B1, B2, B3, B5, B6, B12, C, and D, iron, calcium, and zinc. There was a positive association between CRC and dietary intake of carbohydrate and vitamin A and a negative association with the intake of fiber, beta carotene, vitamin E, folate, and biotin.

In line with the present study, some studies reported that calorie and carbohydrate intake are associated with an increased risk of cancer, and carbohydrate restriction may have a protective effect against cancer (Lv et al., 2014). The ketogenic diet (KD) is a low carbohydrate diet and was reported to be beneficial in patients with CRC (Zhu et al., 2019). However, cancer cells often have high reactive oxygen species that are involved in tumor growth (Stafford et al., 2010) and KD may increase reduced reactive oxygen which has adverse effects on several cancer‐related pathways such as apoptosis and metastasis (Weber et al., 2020).

Regarding dietary fiber, some studies reported an inverse association between colorectal cancer and dietary fiber intake (Bingham et al., 2003; Kunzmann et al., 2015; Larsson et al., 2005), which was in line with the result of the present study. One meta‐analysis found that whole grains and cereal fiber are negatively associated with CRC (Aune et al., 2011). Moreover, Kok et al. found that higher fiber intake was associated with reduced recurrence in patients with colorectal cancer (Kok et al., 2021). Dietary fibers produce beneficial metabolites in the gut through bacterial fermentation. Consumption of fibers increases the production of beneficial intestinal bacteria such as Bifidobacterium and Eubacterium which leads to an increase in the production of short‐chain fatty acids (SCFAs) including butyrate, acetate, and propionate. These SCFAs may protect the intestine against cancer. Improvements in fiber fermentation in the colon with *L. acidophilus* as a probiotic may reduce the risk of colorectal cancer through decreased inflammatory and apoptotic pathways (Lee et al., 2020). Butyrate acts against human colon cancer cell proliferation via cell cycle arrest and apoptosis (Zeng et al., 2020). Moreover, butyrate has a key role in histone deacetylase inhibition which is currently considered as monotherapy or combination therapy against CRC (Holscher et al., 2015; Schatzkin et al., 2000; So et al., 2018; Tampakis et al., 2014). However, some studies reported inconsistent results on the association between CRC and dietary fiber (Schatzkin et al., 2000; Song et al., 2015). This inconsistency may be due to diversity in fiber sources, differences in tumor types, and large variations in the amount of fiber intake (Zeng et al., 2020).

This study reported that the case group had a higher intake of vitamin A, vitamin K, fluoride, and molybdenum and a lower intake of vitamin E, folate, Se, and Mn than the control group. Zhang et al reported that vitamin E intake could reduce colorectal cancer risk through the genetic pathways (Zhang et al., 2021). Consist with this study, several studies demonstrated that higher intake of vitamin E could be associated with reduced cancer risk (Cui et al., 2018; Zhu et al., 2017). However, one study reported that vitamin E may increase tumorigenesis in the early stages of prostate cancer (Njoroge et al., 2017). Vitamin E may exert key antitumor effects in cancer cells through antiproliferative effects and decreasing metastases of the malignant cells (Montagnani Marelli et al., 2019). In line with this study, some studies reported that folate deficiency could be associated with colorectal cancer risk (Jokić et al., 2011; Kim, 2006). Folate deficiency reduces the availability of methyl group and conversion of homocysteine to methionine. Hyperhomocysteinemia inhibits DNA methylation which may increase the risk of cancer (Gholamalizadeh et al., 2019).

The present study found a positive association between vitamin A and CRC. In contrast with this study, Park et al. reported that vitamin A supplementation reduced hepatic metastases of colon tumor cells in mice (Park et al., 2012). Another study suggested that retinyl palmitate as a dietary retinoid reduced melanoma metastasis in mice (Weinzweig et al., 2003). The exact mechanism of the interaction of vitamin A and colorectal cancer is not fully understood. But the amount of dietary carotenoids may influence the associations between CRC and vitamin A. An inverse association was also found between intake of beta carotene and the risk of CRC. Findings in this study are consistent with the hypotheses that dietary carotenoid intake may reduce the risk of colorectal cancer (Lu et al., 2015). Beta carotene is a member of the carotenoid family, and as a fat‐soluble pigments is naturally present in many fruits, grains, oils, and vegetables (Rao & Rao, 2007). A case–control study in the USA reported that high beta carotene intake was associated with a reduced risk of colorectal cancer (Williams et al., 2010). Another study showed that dietary beta carotene intakes were inversely related to colorectal adenoma (Jung et al., 2013). However, some case–control studies reported no associations for intakes of different carotenoids with colorectal cancer risk (Kune & Watson, 2006; Nkondjock & Ghadirian, 2004; Wang et al., 2012). The inconsistent results may be due to different bioavailability of carotenoids which can vary depending on the cooking method (Gomes et al., 2013; Hwang & Kim, 2013).

Regarding the association between CRC and dietary elements, some studies reported that minerals concentration is different in cancerous and noncancerous tissues, suggesting that these elements may play a vital role in the development of cancer (Sohrabi et al., 2018). The less risk of colorectal cancer among those who eat higher amounts of fruits and vegetables may be partially attributed to magnesium (Polter et al., 2019). Magnesium is required in over 300 enzyme systems that are critical for multiple cellular functions and a low concentration of Mg increases the risk of various diseases (Vormann, 2016). In the present study, the case group had less magnesium intake compared to the control group. The association between dietary magnesium and colorectal cancer risk has been examined in various studies. There is compelling evidence that magnesium intake may significantly protect against CRC (Hou et al., 2013). Calcium also has a beneficial effect against colon cancer and may have shared metabolic pathways with magnesium (Bostick et al., 1993; Zheng et al., 1998). There is strong evidence that consuming dairy products and calcium supplements decrease and consuming red meat and alcoholic drinks increase the risk of CRC (Vingeliene et al., 2017).

The effect of dietary micronutrients on CRC was controversial in recent years. A study from Southeast Siberia showed no association between the consumption of fruits and vegetables and the risk of developing colorectal cancer (Zhivotovskiy et al., 2012). Other factors such as sex, age, tumor location, physical activity, and the intestinal microbiome likely also influence the association between diet and CRC risk (Key et al., 2009). In this study, selenium intake in the case group was significantly lower than in the control group, and zinc intake was not significantly different between the two groups. Consistent with these results, a recent study showed that intake of selenium was inversely associated with CRC risk and dietary zinc was not associated with CRC risk (Luo et al., 2021). Higher levels of reactive oxygen species (ROS) are observed in CRC tissue than in adjacent tissues (Keshavarzian et al., 1992). The antioxidant enzymes glutathione peroxidase and superoxide dismutase are an essential part of the defense system against the accumulation of ROS in all cellular and extracellular compartments and their activities are mainly dependent on selenium (Hansen et al., 2009), zinc (Davis et al., 2000), and manganese (De Rosa et al., 1980) intake.

Eventually, the results of the present study indicated that CRC patients had an inadequate daily intake of key nutrients that protect against CRC risk, vitamin E, fiber, folate, and selenium. There are several epidemiological studies that have shown that a specific diet can enhance or protect against CRC. The worldwide heterogeneity in CRC incidence is strongly suggestive of etiological involvement of environmental exposures, especially lifestyle and diet (Murphy et al., 2019). However, this study had some limitations. Case–control studies are vulnerable to measurement errors in dietary assessments, and selection and recall bias that is likely to lead to a fake relationship. Further experimental studies are needed to explore the effect of dietary components on the risk of colorectal cancer.

## 5 CONCLUSIONS

In this study, the patients with colorectal cancer had a higher intake of calories, carbohydrates, vitamin A, vitamin K, fluoride, and molybdenum and a lower intake of vitamin E, beta carotene, biotin, folate, magnesium, selenium, manganese, and fiber. There was no significant difference in the mean consumption of vitamins B1, B2, B3, B5, B6, B12, C, and D, iron, calcium, and zinc. Further experimental studies are needed to explore the effect of these nutrients on the risk of colorectal cancer and to identify the underlying mechanisms.

## ETHICAL STATEMENT

This study has been approved by the local ethics review boards at Shahid Beheshti University of Medical Sciences (IR.SBMU.CRC. REC.1398.028). The patients/participants provided their written informed consent to participate in this study.

## Data Availability

The raw data supporting the conclusions of this article will be made available by the authors, if they are requested, to any qualified researcher.
